# Novel Psychopharmacological Herbs Relieve Behavioral Abnormalities and Hippocampal Dysfunctions in an Animal Model of Post-Traumatic Stress Disorder

**DOI:** 10.3390/nu15173815

**Published:** 2023-08-31

**Authors:** Hee Ra Park, Mudan Cai, Eun Jin Yang

**Affiliations:** Department of KM Science Research, Korea Institute of Oriental Medicine (KIOM), Daejeon 34054, Republic of Korea; hrpark0109@kiom.re.kr (H.R.P.); mudan126@kiom.re.kr (M.C.)

**Keywords:** post-traumatic stress disorder, herbal medicine, hippocampus, dentate granule cells, neurogenesis

## Abstract

Post-traumatic stress disorder (PTSD) is an anxiety disorder caused by traumatic or frightening events, with intensified anxiety, fear memories, and cognitive impairment caused by a dysfunctional hippocampus. Owing to its complex phenotype, currently prescribed treatments for PTSD are limited. This study investigated the psychopharmacological effects of novel COMBINATION herbal medicines on the hippocampus of a PTSD murine model induced by combining single prolonged stress (SPS) and foot shock (FS). We designed a novel herbal formula extract (HFE) from *Chaenomeles sinensis*, *Glycyrrhiza uralensis*, and *Atractylodes macrocephala*. SPS+FS mice were administered HFE (500 and 1000 mg/kg) once daily for 14 days. The effects of HFE of HFE on the hippocampus were analyzed using behavioral tests, immunostaining, Golgi staining, and Western blotting. HFE alleviated anxiety-like behavior and fear response, improved short-term memory, and restored hippocampal dysfunction, including hippocampal neurogenesis alteration and aberrant migration and hyperactivation of dentate granule cells in SPS+FS mice. HFE increased phosphorylation of the Kv4.2 potassium channel, extracellular signal-regulated kinase, and cAMP response element-binding protein, which were reduced in the hippocampus of SPS+FS mice. Therefore, our study suggests HFE as a potential therapeutic drug for PTSD by improving behavioral impairment and hippocampal dysfunction and regulating Kv4.2 potassium channel-related pathways in the hippocampus.

## 1. Introduction

Post-traumatic stress disorder (PTSD), which has a lifetime prevalence of 3.9% worldwide, is classified as an anxiety disorder with mental health problems that can occur after a severely traumatic event [1]. The key symptoms of PTSD may include flashbacks, nightmares, and severe anxiety, as well as uncontrollable thoughts about the traumatic event [2]. Those symptoms are closely associated with hippocampal dysfunction, which is a key contributor to PTSD features, including trauma-related experience, overgeneralization of fear responses, and spatial working memory loss [3]. Structural magnetic resonance imaging studies reported that the hippocampal volume of patients with PTSD, particularly in the dentate gyrus (DG) and CA3 hippocampal subregions, is lower than that of healthy participants or patients with social anxiety disorder [4,5,6,7]. Moreover, evidence indicates that constant stress may damage the hippocampus by releasing the stress hormone cortisol [8,9]. Therefore, structural and functional changes in hippocampal neurons, including DG, may be essential factors contributing to the pathological symptoms and behavioral phenotypes of PTSD.

Currently, selective serotonin reuptake inhibitors (SSRIs), such as sertraline, paroxetine, and fluoxetine, as well as selective serotonin-norepinephrine reuptake inhibitors and mood stabilizers, are frequently prescribed for patients with PTSD to help relieve mood- and anxiety-related symptoms [10,11]. However, these medications are only partially effective in some patients with PTSD and are accompanied by side effects, such as decreased appetite, diarrhea, headaches, memory problems, and insomnia [12]. Moreover, PTSD is a highly heterogeneous disorder characterized by complex features. Therefore, current treatments for PTSD are limited, and more effective novel treatments must be explored.

The use of complementary and alternative medicines containing pharmacologically active compounds to treat various diseases, such as cancer, neurological, and immune diseases, is increasing worldwide [13,14,15,16,17]. In addition, a combination of herbal medicines can yield synergistic effects and minimize side effects, resulting in enhanced benefits in the treatment of various diseases [18,19,20,21]. The medicinal herbs *Chaenomeles sinensis*, *Glycyrrhiza uralensis*, and *Atractylodes macrocephala* are well known for their neuroprotective, anti-inflammatory, antioxidant, and anticancer activities [22,23,24,25]. The neuroprotective effect of *Chaenomeles sinensis* has been studied in a murine model of trimethyltin-induced memory deficits through increased choline acetyltransferase activity and attenuated learning and memory abilities [26]. *Glycyrrhiza uralensis* and its active components, such as liquiritin, isoliquiritin, and isoliquiritigenin, reportedly exhibit neuroprotective efficacy in β-amyloid-injected mice [27,28,29,30,31,32]. *Atractylodes macrocephala* and its active components, including atractylenolide-III, exert anti-apoptotic and anti-inflammatory effects in hypoxic neurons and ischemia-damaged mouse microglia, respectively [33,34]. In addition, atractylenolide-III exhibits antidepressant- and anxiolytic-like effects in chronic unpredictable mild-stress rat models [35].

Therefore, the present study aimed to develop a novel herbal formula extract (HFE) comprising *Chaenomeles sinensis*, *Glycyrrhiza uralensis*, and *Atractylodes macrocephala*. We investigated whether the use of this new herbal combination could offer a more effective treatment of PTSD. This study demonstrated that the novel HFE alleviated PTSD-like behavioral symptoms and improved hippocampal dysfunctions, suggesting that HFE modulates the functional impairments in the hippocampus of the mice model of PTSD.

## 2. Materials and Methods

### 2.1. Preparation of HFE

Herbal medicines, including *Chaenomeles sinensis*, *Glycyrrhiza uralensis*, and *Atractylodes macrocephala*, were purchased from Kwangmyungdang Medicinal Herbs Co. (Ulsan, Republic of Korea). Water-based herbal formula extraction of the three medicinal herbs (mixed at a 1:1:1 ratio) was outsourced to KOC BIOTECH (Daejeon, Republic of Korea). Herbs (50 g) were extracted with distilled water (1000 mL) at 40 °C in a shaking incubator for 24 h, filtered through Whatman filter paper (Grade #1, 11 μm pore size), and concentrated under reduced pressure. Water extracts were then freeze-dried to obtain a powdered extract, which was freshly dissolved in 0.9% physiological saline before use.

### 2.2. Establishment of PTSD Mice Model

The experimental protocol was approved by the Institutional Animal Care Committee of the Korea Institute of Oriental Medicine (Approval No.: #21-112), and all experiments were performed in accordance with the guidelines of the US National Institutes of Health guidelines and the Animal Care and Use Committee at KIOM.

**Figure 1:**
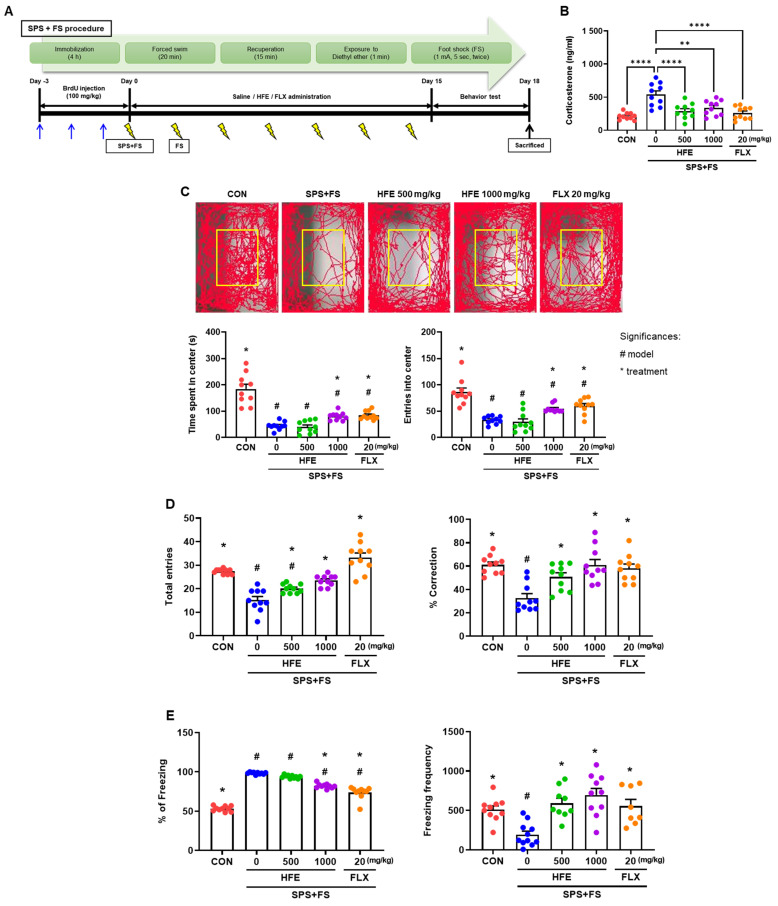
HFE administration ameliorates PTSD-like behaviors in mice in the SPS+FS group. Mice were subjected to behavioral tests after drug administration for 14 days. (**A**) In vivo experimental scheme. (**B**) Serum corticosterone concentrations detected by ELISA. ** *p* < 0.01, **** *p* < 0.0001. (**C**) Movement traces of mice in the open field test. Times and entries in the center zone of the open field test. (**D**) Total number of entries and the percentage of spontaneous alternation in three arms in the Y-maze test. (**E**) Percentage of freezing and freezing frequencies in the fear response test. Data are expressed as the mean ± SEM (*n* = 10 mice/group); Significances: #; significantly different from CON, # *p* < 0.05, *; significantly different from SPS+FS, * *p* < 0.05. CON; control mice, FLX; fluoxetine, FS; foot shock, HFE; herbal formula extract, PTSD; post-traumatic stress disorder, SPS; single prolonged stress.

Male C57BL/6J mice (6 weeks old) were purchased from Daehan BioLink (Eumseong-gun, Chungcheongbuk-do, Republic of Korea). All mice were acclimatized for 7 days before the beginning of the experiments. Mice were maintained under temperature- and light-controlled conditions (20–23 °C, 12/12 h light/dark cycle) with food and water provided ad libitum. Mice were housed in groups of 5 mice per cage. In this study, we developed a mouse model of PTSD based on the single prolonged stress (SPS) protocol, a murine model of PTSD widely used in previous research [36,37]. Mice received SPS, a combination of stressors that included physical restraint for 4 h, and then were immediately exposed to forced swimming in water (diameter: 24 cm, height: 50 cm, water temperature: 24 °C) for 20 min. Mice were then exposed to diethyl ether until they lost consciousness after recuperation for 15 min. Finally, mice received electric foot shock (FS; 1 mA for 5 s, twice every 2 days) after recuperation for 1 h in the shock chamber (Startle and Fear combined system, Harvard Apparatus, Holliston, MA, USA). The in vivo experimental design is summarized in Figure 1A.

### 2.3. Drug Administration

Mice were randomly divided into five groups: (1) control (CON, unstressed mice, *n* = 10 mice), (2) SPS+FS (stressed mice, *n* = 10 mice), (3) SPS+FS+HFE 500 mg/kg (*n* = 10 mice), (4) SPS+FS+HFE 1000 mg/kg (*n* = 10 mice), and (5) SPS+FS+fluoxetine (FLX; Sigma-Aldrich, St. Louis, MO, USA) 20 mg/kg (*n* = 10 mice). All drugs were dissolved in 0.9% physiological saline, and HFE and FLX were given once a day by oral gavage (intragastric administration; IG) for 14 days. Control and SPS+FS groups were administered an equal volume of 0.9% physiological saline by oral gavage (IG). To evaluate the survival of newborn cells in the dentate gyrus (DG) of the hippocampus, mice in each group were administered 6 injections of BrdU (100 mg/kg, i.p., twice daily for 3 days), a marker of proliferative cells in the S-phase, for 3 consecutive days before the experiment.

### 2.4. Open Field Test (OFT)

OFT was used to measure the spontaneous locomotor activity and anxiety level of mice. The equipment for the OFT consisted of a black plastic box (100 cm × 100 cm × 40 cm) with a white bottom and a camera (above the center of the arena). Mice were placed in the center of the box and allowed to explore freely for 20 min. The total distance moved, time spent in the center zone, and entries into the center zone were recorded with image-tracking software. The OFT was performed and analyzed using an EthoVision XT video-tracking software (Noldus, Wageningen, The Netherlands). The equipment was cleaned using 70% ethanol and dried after each trial.

### 2.5. Y-Maze Test

The Y-maze test was used to assess the short-term memory of the mice. The Y-maze apparatus was 40 cm long, 3 cm wide, and 15 cm high, with arms positioned at 120° angles from each other. All mice were placed in the center of the maze and allowed to explore freely for 8 min, and all trials were recorded. A spontaneous alternation was defined as entry into three different arms consecutively (i.e., ABC, CAB, BCA, BAC, or CBA). The percentage of spontaneous alternation was calculated as [(number of alternations/total arm entries − 2)] × 100 (%). The apparatus was cleaned using 70% ethanol and dried after each trial.

### 2.6. Fear Response Test

After finishing the Y-maze test (on day 16), mice, including CON-unstressed mice, were given an electric FS (1 mA for 5 s, twice) in the shock chamber. The next day, mice were exposed to the same environment for 5 min without FS. The total freezing time and freezing frequency of each mouse within 5 min were recorded. Freezing frequency was counted when the freezing duration was 2 s or longer. The apparatus was cleaned using 70% ethanol and dried after each trial.

### 2.7. Tissue Preparation

On day 18, mice were anesthetized with avertin (2,2,2-tribromoethanol, Sigma-Aldrich). For corticosterone assay, serum was collected by centrifugation at 3000 rpm for 15 min at 4 °C. For Western blot analysis, hippocampi were homogenized in RIPA buffer (MilliporeSigma, Burlington, MA, USA), protease inhibitor cocktail, and phosphatase inhibitor cocktail (Roche, Mannheim, Germany) and centrifuged (12,000 rpm for 15 min at 4 °C). The serum and hippocampi lysates were stored at −80 °C until further analyses. For histological analyses, mice were anesthetized with tribromoethanol (Avertin^®^, 240 mg/kg) and perfused intracardially with 4% PFA in 0.1 M phosphate-buffered saline (PBS; pH 7.4). After fixative perfusion, the brains were removed, placed in the same fixative at 4 °C overnight, and transferred to a 30% sucrose solution. Cryoprotected brains were sectioned coronally at 40 μm intervals through their rostral–caudal extent on 1-in-6 series of sections (between −2.80 mm and −5.80 mm posterior to bregma) using a freezing microtome (Leica Biosystems, Wetzlar, Germany) based on Franklin and Paxinos’s stereotaxic mouse brain atlas. For each immunostaining, a single series of six sections (spaced 240 μm apart) was selected from the six available series (1st–6th section per mouse). All sections, including the hippocampus, were collected in Dulbecco’s PBS (DPBS) containing 0.1% sodium azide and stored at 4 °C.

### 2.8. Serum Corticosterone Measurement

Corticosterone levels were determined using an ELISA assay kit (#EIACORT, Thermo Fisher Scientific, Waltham, MA, USA), according to the manufacturer’s instructions. Briefly, 50 μL of standards or samples were added in duplicate to the appropriate wells. Next, 25 μL of corticosterone conjugate and 25 μL of corticosterone antibody were added to each well (except in the nonspecific binding wells). The plate was incubated for 1 h at room temperature with shaking. After the plate was washed and dried, 100 μL of the tetramethylbenzidine (TMB) substrate was added to each well and incubated for 30 min at room temperature. After the reaction was terminated by adding 50 μL of the stop solution, the optical density (O.D.) of corticosterone was read at 450 nm wavelength using a microplate reader (SpectraMax i3; Molecular Devices, San Jose, CA, USA). The corticosterone concentration was calculated according to the standard curves.

### 2.9. Immunostaining

For proliferating cell nuclear antigen (PCNA) or BrdU staining, brain sections were processed, and DNA was denatured by sequentially exposing cells to heat (65 °C), acid (2 M HCl), and base (0.1 M borate buffer) treatments. Brain sections were blocked with tris-buffered saline (TBS)/0.1% Triton X-100/3% bovine serum albumin (BSA) and incubated overnight with primary antibodies at 4 °C. Brain sections were incubated with the avidin-biotin complex (ABC) solution for 1 h at room temperature, washed with TBS, and incubated with biotinylated secondary antibodies (1:300, Vector Laboratories Inc., Newark, CA, USA) for 3 h at room temperature. All cell counts were performed by an investigator (H.R.P.) blinded to the treatment groups. Images were acquired under an optical microscope (BX53, Olympus, Tokyo, Japan). For double-label immunostaining, brain sections were blocked with TBS/0.1% Triton X-100/3% BSA and incubated with primary antibodies overnight at 4 °C. Brain sections were then washed with TBS and incubated with IgG-labeled secondary antibodies with Alexa Fluor (1:300, Vector Laboratories) for 2 h. Confocal z-stack images were acquired using an FV10i FLUOVIEW confocal scanning microscope (Olympus). Only merged cells within the granule cell layer (GCL) were counted. The specific primary antibodies used in this study were PCNA (1:500, mouse, #2586; Cell Signaling Technology, Danvers, MA, USA), BrdU (1:400, rat, #ab6326, Abcam, Cambridge, UK), doublecortin (DCX, 1:500, goat, #sc-8066; Santa Cruz Biotechnology, Santa Cruz, CA, USA), c-fos (1:400, mouse, #sc-166940, Santa Cruz Biotechnology), Prox1 (1:500, rabbit, #ab199359, Abcam), and phosphorylated (p)-CREB (Ser133, 1:500, rabbit, Abcam). Quantitative analysis of histological data was performed by investigators blinded to the results of all images.

### 2.10. Golgi Staining

Golgi staining was performed using the FD Rapid GolgiStain^TM^ kit (#PK401; FD NeuroTechnology, Columbia, MD, USA), according to the manufacturer’s instructions. Briefly, after HFE administration with SPS+FS for 14 days, the brains were obtained via cervical dislocation under CO_2_ anesthesia. Brains were immersed in the impregnation solution for 2 weeks in the dark and transferred into solution C for 1 week. Cryoprotected brains were sectioned coronally at 100 μm intervals through their rostral–caudal axis and mounted on adhesive-coated slides. Sections were stained in a development solution. Images were acquired using a biological microscope (Olympus). The number of dendritic spines was analyzed in a total of 30 dendrites (6 dendrites/mouse; a total of 5 mice/group).

### 2.11. Western Blot Analysis

Hippocampi homogenates were solubilized in SDS-polyacrylamide gel electrophoresis sample buffer, and protein concentrations were determined using a BCA assay kit (Thermo Fisher Scientific) with a BSA standard. Samples (20 μg protein per lane) were separated on SDS-polyacrylamide gels and transferred by electrophoresis to polyvinylidene difluoride transfer membranes (Bio-Rad Laboratories, Hercules, CA, USA). Membranes were blocked with TBS/0.2% Tween-20/5% skim milk and then incubated with primary antibodies overnight at 4 °C. Membranes were washed with TBS and incubated with horseradish peroxidase-conjugated secondary antibodies (1:5000, Thermo Fisher Scientific) for 2 h. Proteins were detected using an enhanced chemiluminescent (ECL) substrate (SuperSignal^TM^ West Femto, Thermo Fisher Scientific) and ChemiDoc Touch Imaging System (Bio-Rad Laboratories). The relative band intensities were calculated using Image Lab software version 6.1.0 (Bio-Rad). Prestained blue protein markers were used to determine molecular weights. The specific primary antibodies used for Western blot analysis were Kv4.2 (1:1000, mouse, #sc-390571, Santa Cruz Biotechnology), phosphorylated (p)-Kv4.2 (Thr602, 1:1000, mouse, #sc-377574, Santa Cruz Biotechnology), p-Kv4.2 (Thr607, 1:1000, mouse, #sc-377545, Santa Cruz Biotechnology), p-ERK (Thr202/Tyr204, 1:1000, rabbit, #4370, Cell Signaling Technology), total (t)-ERK (1:1000, rabbit, #9102, Cell Signaling Technology), and α-tubulin (1:5000, mouse, #MA5-31466, Thermo Fisher Scientific).

### 2.12. Statistical Analysis

Data were evaluated using a one-way analysis of variance and Dunnett’s test. Analyses were performed using GraphPad PRISM software^®^ (GraphPad PRISM software Inc., Version 9.4.1, La Jolla, CA, USA). The results are expressed as the mean ± standard error of the mean. Statistical significance was set a *p* < 0.05.

## 3. Results

### 3.1. Effects of HFE Administration on Serum Corticosterone Levels in PTSD Mice

In this study, we established a murine model of PTSD by exposing mice to several stresses (SPS+FS) and administered HFE (500 and 1000 mg/kg) or FLX for 14 days. FLX is an SSRI that has been widely used in PTSD preclinical research, as well as in clinical patients with PTSD [38,39,40,41]. To verify the successful establishment of the model, we analyzed the serum corticosterone levels of the mice. Serum corticosterone concentration was higher in mice in the SPS+FS group than in CON mice; however, treatment with HFE and FLX significantly suppressed serum corticosterone levels in mice in the SPS+FS group (Figure 1B).

### 3.2. HFE Administration Attenuates PTSD-Like Behavioral Abnormalities

After 14 days of treatment with HFE or FLX, we performed an OFT, Y-maze test, and fear response test to investigate whether HFE can alleviate SPS+FS-induced behavioral abnormalities, such as anxiety, short-term memory deficit, and fear response. Mice in the SPS+FS group showed reduced locomotor activity compared to those in the CON group, as evidenced by a decrease in the time spent and frequency entry into the center zone of the maze, indicating that stressed mice exhibited anxiety-like behavior (Figure 1C). Treatment with 1000 mg/kg of HFE significantly increased the time spent and the number of entries in the center zone. However, there were no significant differences between the SPS+FS group and mice treated with 500 mg/kg HFE. Consistent with reduced locomotion in the OFT, mice in the SPS+FS group showed a reduced total number of entries into all three arms of the Y-maze (Figure 1D). However, treatment with HFE increased the number of total entries into the arms in the Y-maze. Moreover, mice in the SPS+FS group showed a significantly reduced percentage of spontaneous alternation by revisiting the recently visited arm compared with those in the CON group; however, this effect was recovered by treatment with HFE (Figure 1D).

To evaluate the effect of the treatment on PTSD-like behavior, such as fear response, FS-induced freezing response was examined using a fear response test. Mice in the SPS+FS group froze more than those in the CON group in the fear context box (Figure 1E). The freezing frequency was analyzed as an episode with a freezing duration exceeding 2 s. Additionally, mice in the SPS+FS group showed a significant decrease in freezing frequency (Figure 1E), indicating that they were completely dominated by stress and fear. Notably, treatment with HFE (500 and 1000 mg/kg) significantly reduced freezing percentage and frequencies in the fear response test (Figure 1E). Moreover, FLX treatment also significantly ameliorated all the above behavioral parameters (Figure 1C–E).

### 3.3. HFE Administration Protects against Abnormal Hippocampal Neurogenesis in PTSD Mice

**Figure 2:**
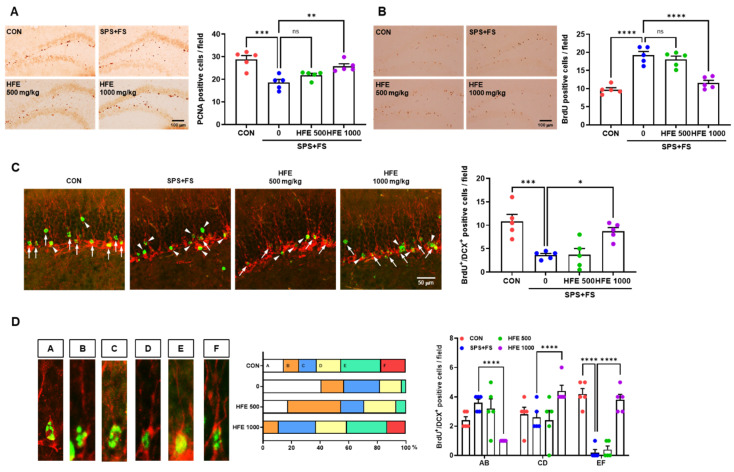
HFE administration prevents abnormal hippocampal neurogenesis in SPS+FS mice. (**A**) Representative images of PCNA (a marker for cell proliferation) in the DG. Scale bar: 100 μm. Quantification of PCNA^+^ cells in the DG of each group. (**B**) Representative images of BrdU (a marker for survival of newborn cells) in the DG. Scale bar: 100 μm. Quantification of BrdU^+^ cells in the DG of each group. (**C**) Representative confocal images of the colocalization of BrdU (green) with DCX (red, a marker for immature DGCs) in the DG of the hippocampus. The arrow indicates co-labeled cells. The arrowhead indicates only BrdU^+^ cells. Scale bar: 50 μm. (**D**) Categorization of dendritic morphology from BrdU^+^/DCX^+^ cells in the DG of the hippocampus. Category A, no processes; Category B, short process; Category C, medium process; Category D, process reaching molecular layer; Category E, one dendrite branching in the molecular layer; Category F, delicate dendritic tree branching in the GCL with quantification of the morphological distribution and number corresponding to each category. Data are expressed as the mean ± SEM (*n* = 5 mice/group); * *p* < 0.05, ** *p* < 0.01, *** *p* < 0.001, **** *p* < 0.0001, ns; not significant. CON; control mice, BrdU; 5-Bromo-2′-Deoxyuridine, DCX; doublecortin, FS; foot shock, HFE; herbal formula extract, PCNA; proliferating cell nuclear antigen, SPS; single prolonged stress; DG, dentate gyrus; DGC, dentate gyrus cells; GCL, granule cell layer.

The hippocampus harmonizes with newborn neurons generated in the subgranular zone (SGZ) and dentate granule cells (DGCs) of the GCL, which is a fundamental region for coordinating memory, including spatial working memory and fear conditioning [2,42,43,44]. Therefore, we investigated the changes in newborn neurons and DGCs in the DG of the hippocampus in mice in the SPS+FS group, as well as the therapeutic effect of HFE treatment in mice with PTSD. To determine the effect of HFE on cell proliferation in the hippocampus, we performed staining with PCNA, a marker of cell proliferation, in the SGZ of the DG. Compared with those in the CON group, PCNA^+^ cells were significantly lower in the SGZ of mice in the SPS+FS group; however, treatment with 1000 mg/kg of HFE restored the number of PCNA^+^ cells (Figure 2A). To assess the effect of HFE on the survival of newborn cells in the hippocampus, BrdU immunostaining was performed. In the DG, we observed an increase in the number of BrdU^+^ cells in the SPS+FS group compared with that in CON group (Figure 2B). Treatment with 1000 mg/kg of HFE decreased the number of BrdU^+^ cells in mice with PTSD (Figure 2B). However, the number of PCNA^+^ or BrdU^+^ cells in the DG of the mice was not significantly affected by treatment with 500 mg/kg of HFE. As indicated by BrdU/DCX immunostaining, the number of newborn immature neurons was significantly lower in the DG of mice in SPS+FS group compared with those in the CON group (Figure 2C). However, treatment with 1000 mg/kg of HFE significantly increased the number of BrdU^+^/DCX^+^ cells. Furthermore, morphological distribution analysis of BrdU^+^/DCX^+^ cells, based on Plümpe’s classification [45], revealed that most BrdU^+^/DCX^+^ cells in the SPS+FS group were distributed in categories AB and CD, with significantly lower numbers of BrdU^+^/DCX^+^ cells belonging to category EF compared with those in CON group (Figure 2D). However, treatment with 1000 mg/kg of HFE significantly increased the number of BrdU^+^/DCX^+^ cells in categories CD and EF. Overall, these results indicate that treatment with HFE restores hippocampal neurogenesis in mice with depression-like symptoms.

### 3.4. HFE Administration Reduces Hilar Ectopic DGCs in PTSD Mice

**Figure 3:**
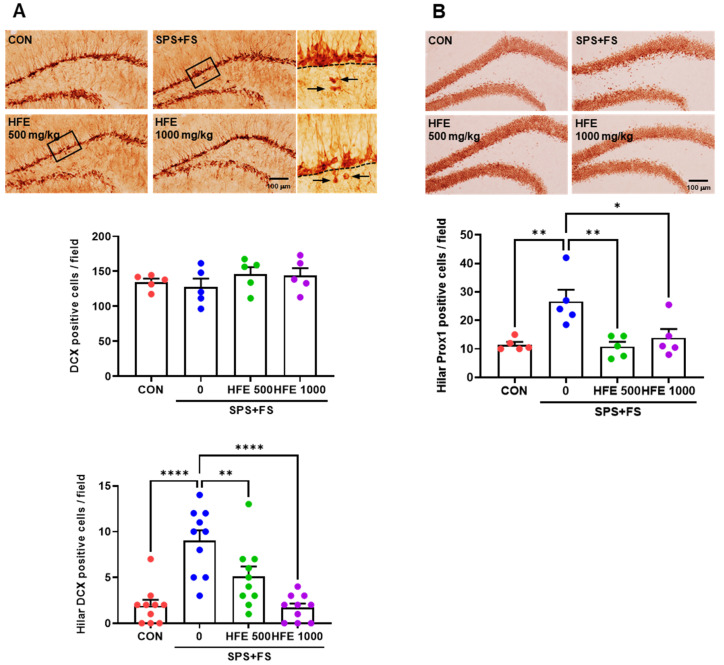
Administration of HFE reduces hilar ectopic DGCs in the DG of SPS+FS mice. (**A**) Representative images of DCX in the DG. Arrows indicate DCX^+^ cells in the hilus. Scale bar: 100 μm. Quantification of DCX^+^ cells in the hilus of the DG of each group (*n* = 10 sections/5 mice/group). (**B**) Representative images of Prox1 (a marker for mature DGCs) in the DG. Scale bar: 100 μm. Quantification of Prox1^+^ cells in the hilus of the DG of each group. Data are expressed as the mean ± SEM (*n* = 5 mice/group); * *p* < 0.05, ** *p* < 0.01, **** *p* < 0.0001. CON; control mice; DCX; doublecortin, FS; foot shock, HFE; herbal formula extract, SPS; single prolonged stress; DG, dentate gyrus; DGC, dentate gyrus cells.

To investigate the effect of SPS+FS-induced stress on DGCs and elucidate the therapeutic effect of HFE, we examined the expression of two DGC markers, DCX and Prox1, using immunostaining. DCX is an early marker expressed in immature DGCs, while Prox1 is a marker expressed in mature DGCs in the DG of the hippocampus [46,47]. There was no significant difference in the number of DCX^+^ cells in the DG between the groups (Figure 3A). However, some DCX^+^ cells were detected in the hilus of the DG in the SPS+FS group, with a significantly higher number of hilar DCX^+^ cells compared with those in the CON group (Figure 3A). Notably, treatment with HFE significantly reduced the SPS+FS-induced increase in the number of hilar DCX^+^ cells in the DG of the mice. In the CON group, most Prox1^+^ cells were identified in the GCL of the DG (Figure 3B). However, hilar Prox1^+^ cells were more abundant in the SPS+FS group than in the CON group; these cells are referred to as “ectopic DGCs” [48,49]. The number of hilar Prox1^+^ cells was significantly reduced by treatment with HFE.

### 3.5. HFE Administration Suppresses SPS+FS-Induced DGC Hyperactivation

**Figure 4:**
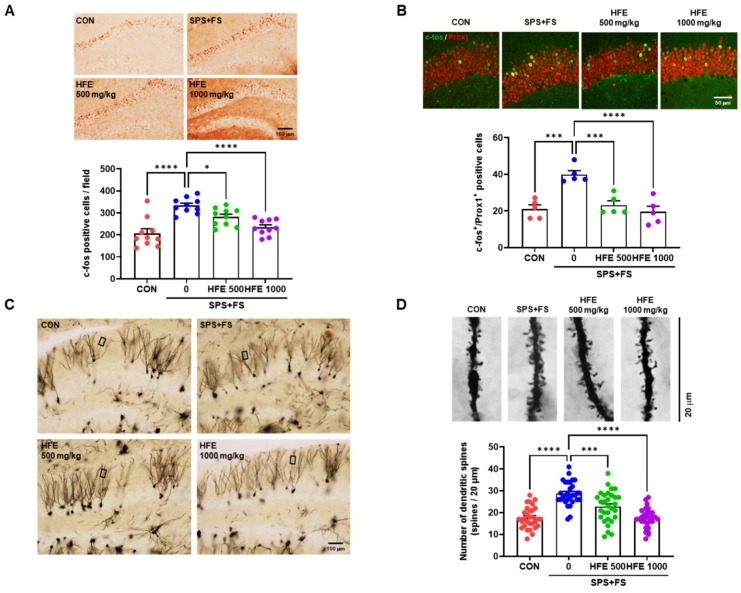
Administration of HFE attenuates the hyperactivation of DGCs and the increase in dendritic spines in the DG of SPS+FS mice. (**A**) Representative images of c-fos (a marker for neuronal activation) in the DG. Scale bar: 100 μm. Quantification of c-fos^+^ cells in the hilus of the DG of each group. (**B**) Representative confocal images of the colocalization of c-fos (green) with Prox1 (red) in the DG of the hippocampus. Scale bar: 50 μm. Quantification of c-fos^+^/Prox1^+^ cells in the DG in each group. Data are expressed as the mean ± SEM (*n* = 5 mice/group). (**C**) Representative images of Golgi staining in the DG. Scale bar: 100 μm. (**D**) Representative images of dendritic spines using Golgi staining. Scale bar: 20 μm. Quantification of the number of dendritic spines of each group. Data are represented as the means ± SEM (*n* = 30 dendritic spines/5 mice); * *p* < 0.05, *** *p* < 0.001, **** *p* < 0.0001. CON; control mice; FS; foot shock, HFE; herbal formula extract, SPS; single prolonged stress; DG, dentate gyrus; DGC, dentate gyrus cells.

To determine the effect of HFE in SPS+FS-induced DGC activity in mice with PTSD, we examined the expression of c-fos, an indicator of the neuronal activity of DGCs in the DG. Compared with that in the CON group, SPS+FS treatment increased c-fos expression and the number of c-fos^+^/Prox1^+^ cells in the DG (Figure 4A,B), indicating that SPS+FS abnormally increased DGC activation. However, HFE treatment significantly reduced the number of c-fos^+^ and c-fos^+^/Prox1^+^ cells in the DG of mice with PTSD (Figure 4A,B). Furthermore, we performed Golgi staining of brain sections, including the DG region, to elucidate the effect of HFE on SPS+FS-induced changes in neuronal dendrites and dendritic spines. There was a significant increase in the number of dendritic spines in the DGCs of mice in the SPS+FS group compared with those in the CON group (Figure 4C,D), which was significantly reduced upon HFE treatment.

### 3.6. HFE Restores the Activation of the Kv4.2/ERK/CREB Pathway Reduced by SPS+FS

**Figure 5:**
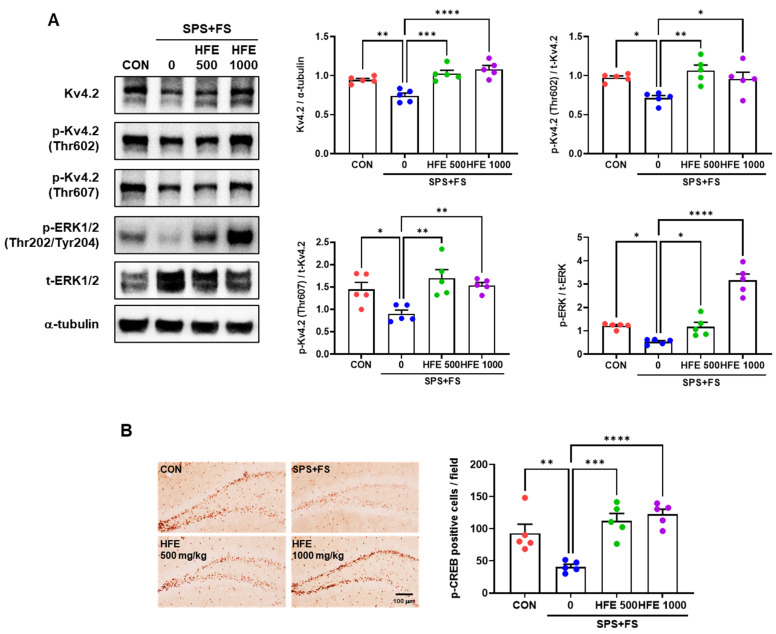
Administration of HFE increases the expression of the Kv4.2/ERK/CREB pathway in the hippocampus of SPS+FS mice. (**A**) Representative bands of Kv4.2, p-Kv4.2 (Thr602), p-Kv4.2 (Thr607), p-ERK (Thr202/Tyr204), t-ERK, and α-tubulin proteins in the hippocampus. Quantification of the ratio of Kv4.2/α-tubulin, p-Kv4.2/t-Kv4.2, and p-ERK/t-ERK by densitometric analysis. (**B**) Representative micrographs of p-CREB (Ser133) in the DG of the hippocampus. Scale bar: 100 μm. Quantification of p-CREB^+^ cells in the SGZ of the DG. Data are presented as the mean ± SEM (*n* = 5 mice/group); * *p* < 0.05, ** *p* < 0.01, *** *p* < 0.001, **** *p* < 0.0001. CON; control mice, CREB; cAMP response element-binding protein, ERK; extracellular signal-regulated kinase, FS; foot shock, HFE; herbal formula extract, SPS; single prolonged stress; DG, dentate gyrus.

Furthermore, we investigated the therapeutic mechanism of HFE in SPS+FS-mediated DGCs activation and increased number of dendritic spines. Kv4.2, a subunit of dendritic transient A-type K^+^ current in neurons, is involved in the regulation of dendritic excitability and plasticity [50,51]. Western blot analysis indicated a decrease in Kv4.2 expression in the hippocampus of mice in the SPS+FS group compared with that in the CON group (Figure 5A). Additionally, there was a significant decrease in Kv4.2 phosphorylation at Thr602 and Thr607 in the SPS+FS group. However, HFE treatment recovered Kv4.2 expression and phosphorylation at Thr602 and Thr607 in the hippocampus of mice in the SPS+FS group. p-ERK can modulate the channel activity by direct phosphorylation of Kv4.2 at Thr602 and Thr607 [52,53]. Therefore, we assessed the phosphorylation of ERK in the hippocampus and found that SPS+FS decreased p-ERK expression, whereas treatment with HFE restored it (Figure 5A). ERK1/2 can also activate CREB by phosphorylation at Ser133, leading to the induction of gene transcription [54]. Immunostaining for CREB phosphorylation at Ser133 indicated that p-CREB^+^ cells were predominantly observed in the SGZ of the DG (Figure 5B), which was consistent with previous findings [55,56]. Moreover, the number of p-CREB^+^ cells was significantly lower in the DG of SPS+FS-treated mice compared with that in the CON group (Figure 5B). Notably, treatment with 500 or 1000 mg/kg HFE prevented SPS+FS-induced reduction in p-CREB expression in the DG.

## 4. Discussion

PTSD is a serious mental disorder triggered by a terrifying event, causing flashbacks, nightmares, and severe anxiety. Currently, a combination of various medications, such as antidepressants, mood stabilizers, and memory improvers, are prescribed to alleviate the various and complex symptoms of patients with PTSD [57]. However, there are limitations to these therapies, such as unwanted side effects or the inability to address the underlying PTSD pathophysiology. Therefore, the development of novel therapeutic drugs for PTSD is necessary. To the best of our knowledge, this study is the first to investigate the effect of a novel HFE in mice with PTSD-like symptoms. HFE ameliorated anxiety, memory loss, and fear response in mice with PTSD-like symptoms. Moreover, the alleviating effects of HFE on PTSD-like symptoms were associated with the rescue of hippocampal dysfunction, including changes in hippocampal neurogenesis, hilar ectopic DGCs, and hyperactivation of DGCs.

Anxiety, spatial working memory loss, and fear-dependent response are key symptoms of PTSD [58]. In the present study, PTSD-related behavior abnormalities were alleviated by treatment with HFE, with reduced freezing time, increased locomotor activity, and spontaneous alternation. These results suggest that HFE could be an effective antidepressant, anxiolytic, and memory-improving drug. Additionally, HFE treatment improved spatial working memory in the Y-maze test and decreased freezing time in the fear response test. Previous reports suggest that anxiety and fear can limit the spatial working memory capacity by competing with task-related processes in the brain [59,60]. Vytal et al. have suggested that high anxiety and fear levels decrease the working memory capacity because more energy is required for anxiety [59]. Therefore, our results indicate that HFE is beneficial for treating PTSD-induced behavioral abnormalities by reducing anxiety and increasing spatial working memory.

The DG subregion of the hippocampus controls anxiety and memory functions by regulating the hippocampal neural circuits through interaction between existing DGCs and newborn neurons generated from hippocampal neurogenesis [2,42,43,44]. Newborn neurons are generated from NSC proliferation, fate determination, and survival of newborn cells through hippocampal neurogenesis processes. Therefore, we determined the modulating efficacy of HFE on hippocampal neurogenesis in SPS+FS-treated mice. HFE effectively modulated abnormal hippocampal neurogenesis in SPS+FS-treated mice, as evidenced by reduced NSC proliferation, increased newborn cell survival, and a decreased number of BrdU^+^/DCX^+^ cells. Consistent with previous reports that PTSD is involved in hippocampal neurodegeneration, reduced neurogenesis, and memory deficits [61,62], our data also show a reduction in hippocampal neurogenesis in mice with PTSD, whereas treatment with HFE dramatically restored the expression of specific markers of hippocampal neurogenesis.

Hilar ectopic DGCs form abnormal neural connections with adjacent DGCs and interfere with hippocampal neural circuits [49,63,64]. Although these cells have been mainly studied in temporal lobe epilepsy, which is characterized by recurrent seizures accompanied by temporarily increased hippocampal neurogenesis, they are also observed in patients with schizophrenia and alcoholism [65,66,67]. In traumatic brain injury, neurons formed after excessive stress such as trauma are localized ectopically to the hilus and molecular layer in the hippocampus [68,69]. Ectopic DGCs are also present in prenatal animals exposed to external stress or injury, such as prenatal isoflurane exposure, methylphenidate, and prenatal inflammation, resulting in the disruption of hippocampal function [70,71,72]. Although there are no reports on the detection of ectopic DGCs in animal models of PTSD, we observed that the levels of DCX^+^ and Prox1^+^ cells in the hilus were significantly increased in mice in the SPS+FS group, which was significantly reduced by HFE treatment. Therefore, our results demonstrate another pathological pattern for hippocampal functional impairment in PTSD, suggesting HFE as a potential new effective drug for PTSD-induced hippocampal functional impairment.

Anxiety is related to the increase in neuronal excitability in the hippocampus and changes in hippocampal neuronal function, underlying the behavioral abnormalities of anxiety-associated disorders [73,74]. Our results also show that mice in the SPS+FS group had high levels of c-fos^+^/Prox1^+^ cells and dendritic spines in the DG, indicating hyperactivation of mature DGCs. In contrast, HFE significantly downregulated SPS+FS-induced hyperactivation of mature DGCs in the DG. Alterations in the balance of excitation/inhibition of mature DGCs are potential targets for mediating hippocampal dysfunction and have clinical value in treating neurological conditions [75]. Hyperexcitability of DGCs by the degeneration of hilar mossy cells induces impaired pattern separation, which is a predictor of anxiety and PTSD [76]. Moreover, various stress signals that trigger the excitability and plasticity of hippocampal neurons have been analyzed through in vivo Ca^2+^ imaging, electrophysiology, and c-fos expression in social defeat stress, chronic restraint stress, and corticosterone-treated mice [77,78,79].

The inhibition of or reduction in voltage-gated potassium (Kv) channels, including Kv4.2, affects neuronal activity by increasing excitability in the hippocampus [80]. Kv4.2 plays a key role in controlling neuronal plasticity and contributing to learning and memory [81,82]. Moreover, mature DGCs rarely fire action potentials and are strongly inhibited by interneurons and ion channels, resulting in low intrinsic excitability and a higher induction threshold for long-term potentiation [83]. Tiwari et al. revealed that mice with heterozygous deletion of Kv4.2 exhibit a reduction in dendritic spine density and high sensitivity to chemoconvulsants, suggesting that Kv4.2 modulates neuronal excitability in the hippocampus [84]. Additionally, Kv4.2 expression is reduced in the hippocampus of chronic mild-stress-exposed rats [85]. In the present study, there was a significant decrease in the expression of Kv4.2 and its phosphorylated form in the hippocampus in mice in the SPS+FS group, which was recovered by HFE treatment. These results suggest that the therapeutic effects of HFE on hippocampal function and PTSD-like behaviors may be mediated through the regulation of the Kv4.2 potassium channel. Kv4.2 is directly phosphorylated by ERK at three sites (Thr602, Thr607, and Ser616) [86]. Moreover, ERK is directly activated by CREB-induced phosphorylation at Ser133 [87]. In the present study, treatment with HFE significantly reversed a SPS+FSS-induced decrease in the expression of phosphorylated ERK and CREB. Overall, these findings suggest that the psychopharmacological mechanism of HFE is mediated through regulation of the Kv4.2/ERK/CREB pathway to restore the hippocampal dysfunction and behavioral abnormalities in SPS+FS-treated mice, suggesting that HFE is a potential treatment for PTSD.

## 5. Conclusions

In this study, we demonstrated that HFE modulated anxiety-like behavior, short-term memory impairment, and freezing response to fear elicited by PTSD. HFE could help restore newborn cells in the DG of the hippocampus of PTSD-like mice. We further found that ectopic migration and hyperactivation of DGCs in PTSD-like mice were reduced by treatment with HFE. Our study suggests that the Kv4.2/ERK/CREB pathway mediates the therapeutic effect of HFE by ameliorating the alterations in the hippocampus of PTSD-like mice. Additionally, there was no significant change in body weight and motor ability between groups, suggesting that HFE administration did not cause side effects or toxicity in the mice. However, it is considered necessary to evaluate the toxicity of HFE through long-term administration studies and pathological analysis of other organs. Therefore, this study suggests the potential of HFE as a therapeutic drug for PTSD and other psychological disorders. Nonetheless, further investigations are needed to evaluate the therapeutic efficacy of HFE on patients with PTSD. Additionally, approaches to the hypothalamic-pituitary–adrenal axis function and hippocampal neural circuits are warranted to clarify the mechanisms underlying the therapeutic efficacy of HFE.

## Data Availability

The datasets supporting the conclusions in this study are contained in the article.
